# Efficient Human Breast Cancer Xenograft Regression after a Single Treatment with a Novel Liposomal Formulation of Epirubicin Prepared Using the EDTA Ion Gradient Method

**DOI:** 10.1371/journal.pone.0091487

**Published:** 2014-03-12

**Authors:** Jerzy Gubernator, Dominik Lipka, Mariola Korycińska, Katarzyna Kempińska, Magdalena Milczarek, Joanna Wietrzyk, Rafał Hrynyk, Sabine Barnert, Regine Süss, Arkadiusz Kozubek

**Affiliations:** 1 Department of Lipids and Liposomes, Faculty of Biotechnology, University of Wroclaw, Wroclaw, Poland; 2 Department of Experimental Oncology, Ludwik Hirszfeld Institute of Immunology and Experimental Therapy Polish Academy of Sciences, Wrocław, Poland; 3 Department of Personal Protective Equipment. Central Institute for Labour Protection - National Research Institute, Warsaw, Poland; 4 Department of Pharmaceutical Technology, Albert-Ludwigs University, Freiburg, Germany; Wayne State University School of Medicine, United States of America

## Abstract

Liposomes act as efficient drug carriers. Recently, epirubicin (EPI) formulation was developed using a novel EDTA ion gradient method for drug encapsulation. This formulation displayed very good stability and drug retention *in vitro* in a two-year long-term stability experiment. The cryo-TEM images show drug precipitate structures different than ones formed with ammonium sulfate method, which is usually used to encapsulate anthracyclines. Its pharmacokinetic properties and its efficacy in the human breast MDA-MB-231 cancer xenograft model were also determined. The liposomal EPI formulation is eliminated slowly with an AUC of 7.6487, while the free drug has an AUC of only 0.0097. The formulation also had a much higher overall antitumor efficacy than the free drug.

## Introduction

Liposomes are one of the best available drug delivery systems. They can favorably alter the pharmacokinetics and pharmacodynamics of encapsulated drugs. The increased liposomal efficacy may easily be correlated with the high drug-to-lipid ratio, the long half-life for encapsulated drugs and the phenomenon of EPR (enhanced permeability and retention), all of which make the concept of Ehrlich's magic bullet at least partially feasible [1]–[4].

Despite the huge potential of liposomes, they have only been applied for a relatively small number of drugs, partially due to the relatively low stability of liposomes but also because of the difficulty of developing stable and efficient drug formulations. Another issue is related to the drug physico-chemical properties, which can make liposome encapsulation difficult or impossible in the case of certain drugs [5]–[7]. Recently, an increasing number of liposomal formulations is entering clinical trials, promising the market appearance of novel formulations of potent anticancer drugs [8]–[12].

Epirubicin (EPI; 4′-epidoxorubicin), a stereoisomer of the anthracycline doxorubicin (DOX), has been clinically applied in treating breast cancer, non-Hodgkin's lymphomas, ovarian cancer, soft-tissue sarcomas, pancreatic cancer, gastric cancer, small-cell lung cancer and acute leukemia [13]. EPI shows less hematologic or myocardial toxicity than DOX at comparable doses and is thus one of the most interesting candidate drugs for liposome encapsulation [14]. Additionally, tumor cells absorb EPI more quickly than DOX, so it is possible to obtain a higher dose of the drug in the tumor cell interior, which increases its potential anticancer activity [15].

Only a few liposomal formulations of the EPI have been reported so far [13], [16]–[18]. Our assessments indicate that the drug has a huge market potential and our goal was to prepare a stable, long-circulating liposomal formulation of EPI for treatment of advanced breast cancer. The drug properties indicate favorable drug behavior in terms of its effective encapsulation within liposomes at a wide range of drug-to-lipid ratios. To achieve this, we applied a new drug encapsulation method developed in our laboratory. This method has proven to yield formulations that give slower drug release for anthracyclines with rapid bilayer permeability, such as idarubicin (IDA) by formation of dense and low soluble EDTA-IDA precipitates inside the liposomes and therefore slowing down drug escape from the liposomes in vivo [19]. In case of much less hydrophobic EPI, the increase of the drug release rate is required to achieve better drug activity in cancer tissue. Fortunately, EDTA-EPI salt has about 50-fold higher solubility compared with EDTA-IDA salt. The increase of drug release at the site of action is one of the postulates of the researchers working with commercially available referential liposomal Doxorubicin formulation Doxil [20], [21]. The direct comparison of the EPI EDTA and sulfate salts favors EDTA salts which have better solubility at low pH. This should have some impact on drug release rate. This hypothesis is supported by the comparison of the drug precipitates in EDTA ammonium salt and ammonium sulfate salt - the structure of the drug precipitate in the EDTA loaded formulation differs from the well-known Doxil - bundle like drug precipitate structure. In our current study, we tested the new EPI formulation obtained using this encapsulation method. During the experiments, no problems related to drug encapsulation or liposome stability were encountered, indicating the potential of the EPI-containing liposomes in breast cancer treatment. Pharmacokinetics studies were performed for both the free drug and liposomal formulation, and drug anticancer activity was assessed using a human breast cancer mouse xenograft model. The liposomal formulation showed a dramatic improvement in anticancer activity with moderate decrease in side toxicity compared to the free drug. Further investigations of this liposomal EPI formulation are under way.

## Materials and Methods

### 2.1. Materials

The hydrogenated soya phosphatidylcholine Phospholipon 100 H (HSPC) was donated by Phospholipid GmbH (Cologne, Germany). 1,2-Distearoyl-sn-glycero-phosphoethanolamine-N-[poly(ethylene glycol)2000] (DSPE-PEG 2000) and cholesterol (Chol) were purchased from Northern Lipids, Inc. (Vancouver, British Columbia, Canada). Sodium dihydrogen phosphate, disodium hydrogen phosphate, sodium chloride, EDTA diammonium salt, HEPES and Sephadex G-50 fine were obtained from Sigma-Aldrich Chemie GmbH (Steinheim, Germany). Epirubicin hydrochloride (EPI) was donated by the Pharmaceutical Research Institute (Warsaw, Poland). All the other reagents were of analytical grade.

### 2.2. Preparation of liposomes

HSPC/Chol/DSPE-PEG 2000 (5.5:4:0.5 mol/mol) liposomes were prepared using the extrusion method. Briefly, 30–90 mg of lipids were dissolved in 4 ml of cyclohexane with 100 μl of methanol and frozen in liquid nitrogen. The sample was then freeze-dried overnight at low pressure using a Savant Modulyo apparatus (Savant, USA). Multilamellar vesicles (MLVs) were formed by hydrating the lipid film with 2–3 ml EDTA diammonium salt solution, pH 4.3, at 64°C, followed by seven freeze-thaw cycles. Large unilamellar vesicles (LUVs) were prepared by extrusion through Nucleopore polycarbonate filters with pore sizes of 100 nm (10 passes) on a Thermobarrel Extruder (Lipex Biomembranes, Vancouver, British Columbia, Canada). The extruder was equilibrated to a temperature of 64°C prior to liposome extrusion. The mean diameter of the vesicles was determined (multimodal analysis, volume weighted) using a Zetasizer Nano-ZS (Malvern Instruments Ltd., Malvern, UK). They were generally in the size range 106–115 nm.

### 2.3. Preparation of the ion gradient for drug encapsulation

The ion gradient was generated by exchanging the extravesicular liposomal solution on Sephadex G-50 (1×20 cm) columns equilibrated with PBS buffer (20 mM sodium phosphate, 150 mM NaCl, pH 7.4). In the collected liposomal fraction, the lipid concentration was then determined using the ammonium ferrothiocyanate assay [22]. The liposomes were then diluted with the same PBS buffer to a final lipid concentration of 10 mM. EPI hydrochloride solution in 150 mM NaCl (6 mg/ml) was added to the LUV suspension to achieve a drug-to-lipid ratio of 1:5 w/w (∼1:4 molar ratios). The loading process was carried out at 60°C for 15 min. For the kinetics of drug encapsulation experiments, 50-μl liposomal samples were taken after loading for 2.5, 5, 15, 30 or 60 min.

### 2.4. Determination of encapsulation efficiency (EE)

Non-encapsulated drug was removed from the EPI-containing liposomes by size exclusion chromatography on a Sephadex G-50 mini-column (5.5×70 mm) equilibrated with 150 mM NaCl solution. Liposome samples (50 μl) were placed on the column and the free EPI was separated from the EPI-containing liposomes. The concentration of the drug in the liposomal samples was assessed photometrically at 482 nm (Shimadzu UV 2401 PC spectrophotometer, Shimadzu, Japan) after solubilization of the liposomes with Triton-X 100 at 60°C. The encapsulation efficiency (EE) was calculated as the percentage of EPI remaining with the liposomes following elution, for the normalized lipid concentration.

### 2.5. The stability of the EPI-loaded liposomes in the presence of plasma

EPI was encapsulated in HSPC/Chol/DSPE-PEG 2000 (mol/mol 5.5:4:0.5) liposomes using a gradient of 300 mM ammonium sulfate (pH 5.5), citrate buffer (pH 4.0) or EDTA diammonium salt (pH 4.3) at the 1:5 weight ratio. Afterwards, non-encapsulated drug was removed by size exclusion chromatography on Sephadex G-50 mini-columns (5.5×70 mm) equilibrated with 150 mM sodium chloride solution. Then the liposomal samples were diluted to obtain a 4 mM liposomal concentration. Two ml of each liposomal suspension were then mixed with 2 ml of fresh human plasma to a final lipid concentration of 2 mM. The liposomes were incubated at 37°C for 24 h. At 0, 1, 3, 6, and 24 h, 100-μl samples were separated from the released drug on Sepharose CL-4B mini-columns. Then the drug and lipid content was measured in the collected liposomal fractions and compared with the initial values. The Sepharose CL-4B columns were selected

### 2.6. Cryo-transmission electron microscopy (Cryo-TEM)

For the cryo-transmission electron microscopy experiments, EPI-loaded liposomes consisting of HSPC/Chol/DSPE-PEG 2000 (mol/mol 5.5:4:0.5) with of 1:5 drug to lipid ratio (w/w) were prepared. The liposomal formulations were prepared to a total lipid concentration of 7–9 mM. Copper grids (Quantifoil S7/2 Cu 400 mesh, holey carbon films, Quantifoil Micro Tools GmbH, Jena, Germany) were prepared according to a standard procedure. After placing a drop of the sample on the grid, most of the liquid was removed with filter paper, leaving a thin film stretched over the holes. The samples were immediately shock-frozen by plunging into liquid ethane, stored at 90K in liquid nitrogen and loaded into a cryogenic sample holder (D262, Gatan Inc, Plesanton, USA). The samples were transferred to the microscope (LEO 912 OMEGA; Carl Zeiss, Oberkochen, Germany) as described elsewhere [23] and then examined at 100 K. Digital images were recorded with a “slow scan CCD camera system” (Proscan HSV 2, Oxford instruments, Abingdon USA under low-dose conditions.

### 2.7. EPI long-term in liposome retention experiments

Liposomes were prepared as described in sections 2.2 to 2.4. Non-encapsulated drug was subsequently removed on Sephadex G-50 mini-columns (5.5×70 mm) equilibrated with a buffer solution containing PBS and 0.1% NaN_3_, pH 7.4. The liposomes (9 mg/ml TL or 1.5 mg EPI/ml) were stored as suspension for 24 months at 4°C. At selected time intervals, 50-μl liposomal samples were placed on Sephadex G-50 mini-columns (5.5×70 mm) equilibrated with PBS buffer (20 mM sodium phosphate, 150 mM NaCl, pH 7.4). The liposomal samples were collected and the amount of EPI was determined fluorometrically on a Cary Eclipse spectrofluorometer (Varian, USA) using an excitation wavelength of 485 nm and an emission wavelength of 595 nm after dissolution of the liposomes in methanol. The lipid concentration was also assessed and the encapsulation efficiency was calculated as described above. The size and polydispersity index of the liposomes were determined on a Zetasizer Nano-ZS (Malvern Instruments Ltd., Malvern, UK), in volume weighting mode.

### 2.8. Plasma elimination of liposomal EPI

HSPC/Chol/DSPE-PEG 2000 5.5:4:0.5 (mol/mol) liposomes containing EPI (drug-to-lipid ratio 1:5 w/w), encapsulated using the EDTA ion gradient method were injected into mice (BALB/c, male, 5 per group, 22–24 g) via the lateral tail vein at a dose of 34.5 μmole/kg body weight (20 mg/kg). At selected intervals, the mice were anesthetized with isoflurane (Forane) and blood was collected from the retroorbital sinus. The blood samples were immediately centrifuged (2000×g, 10 min, 4°C) to separate the plasma and erythrocytes. For HPLC determination, 100 μl of plasma samples were frozen for 12 h and then deproteinized with 100 μl of acetonitrile. The samples were mixed, vortexed for 2 min, and then centrifuged (25,000×g, 5 min, 25°C). The supernatant was injected onto the column.

The concentration of EPI was determined by HPLC analysis. This method employed a Waters 660 Pump, an XTerra RP18 column (250 mm×4.6 mm, 5 μm), and a Water/Acetonitrile/Tetrahydrofuran/H_3_PO_4_/Triethylamine (312:165:20:1:2, v/v) mobile phase with a flow rate of 1 ml/min. The detection was done using a Waters 474 Scanning Fluorescence Detector at an excitation wavelength of 482 nm and an emission wavelength of 542 nm. Ten μl of each sample was injected on the chromatographic column and separated at 25°C. A Waters Millennium Version 3.20 processing module was used to record and process the chromatograms. The relative amount of EPI was calculated from the appropriate calibration curve in the range from 0.25–20 μg/ml.

This method was validated for liposomal EPI mixed with human plasma and applied as described above with a recovery efficiency of 92–104%. Values for the pharmacokinetic parameters of all formulations (AUC, T_1/2_, AUMC, MRT, Vdss and Clb) were calculated by non-compartmental analysis (n = 5) using WinNonlin Software, version 5.1 (Pharsight Inc., Mountain View, CA, USA). [24].

### 2.9. Liposomal EPI efficacy in the human breast MDA-MB-231 cancer xenograft model

Compounds. EPI and liposome formulations with or without EPI were prepared in PBS buffer as described above and administered to mice at a volume of 10 μl/g body weight.

Cell lines. The MDA-MB-231 human breast adenocarcinoma cell line was donated by the Fibiger Institute, Copenhagen, Denmark. It was cultured *in vitro* in the Cell Culture Collection of the Institute of Immunology and Experimental Therapy, Wroclaw, Poland. The cell line was cultured *in vitro* in RPMI-1640 medium (IIET, Wroclaw, Poland) supplemented with 2 mM L-glutamine (Sigma-Aldrich), 10% fetal bovine serum (Thermo Scientific), 100 U/ml penicillin and 100 μg/ml streptomycin (both from Polfa Tarchomin S.A. Warsaw, Poland). The cells were cultured at 37°C in a humid atmosphere saturated with 5% CO_2_.

The MCF-7 human breast cancer cells (Institute of Immunology and Experimental Therapy, Wroclaw, Poland) were cultured in MEM Eagle (Institute of Immunology and Experimental Therapy, Wroclaw, Poland) supplemented with 10% fetal bovine serum and 2 mM glutamine (all reagents were from Sigma-Aldrich), 100 U/ml penicillin, 100 μg/ml streptomycin and 25 μg/ml Amphotericin B (all from Polfa Tarchomin S.A. Warsaw, Poland). The cells were cultured at 37°C in humid atmosphere saturated with 5% CO_2_.

Mice. NOD/SCID female mice (weighing 18–23 g) were obtained from the University Children's Hospital, Cracow, Poland, and maintained in specific pathogen-free conditions.

The work described in this article have been carried out in accordance with The Code of Ethics of the World Medical Association (Declaration of Helsinki) for experiments involving animals, and approved by the 1^st^ Local Committee for Experiments with the Use of Laboratory Animals, Wroclaw, Poland.

Details of the treatment schedules. The MDA-MB-231 human breast adenocarcinoma cells derived from *in vitro* culture were inoculated subcutaneously (s.c.) in the right flank region into NOD/SCID mice at a quantity of 8×10^6^ cells suspended in 0.2 ml saline per mouse (day 0). On the 6^th^ day after tumor cell inoculation, the mice were randomly divided into control and treated groups (from 5 to 8 mice per group) and then injected once intravenously with EPI or with a liposome formulation with or without EPI. The EPI and liposomes with EPI were administered to the mice in two different doses of 6 and 9 mg/kg. Empty liposomes were administered in the doses used to prepare the liposome formulations with EPI. The control group received saline. Evaluation of the therapeutic effect. Tumor volume was calculated using the formula 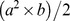
where a was the shorter tumor diameter in mm and b was the longer tumor diameter in mm.

The inhibition of tumor growth was calculated from the formula 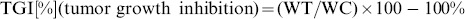
where WT was the median tumor volume of the treated mice and WC was that of the untreated control animals.

The antitumor effect *in vivo* was also evaluated as the increase in life span (ILS) of treated mice over the control, calculated from the formula 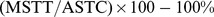
where MSTT was the median survival time of treated animals, and MSTC was the median survival time of untreated control mice.

Body weight changes. The average body weight change (BWC) in all groups was calculated using the formula
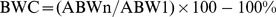
where ABWn is the average body weight on the n^th^ day of the experiment (during treatment) and ABW1 is the average body weight on the first day of treatment.

Statistical evaluation. Statistical analysis was performed using STATISTICA version 7.1 (StatSoft, Inc., USA). For tumor growth inhibition analysis, Kruskal-Wallis ANOVA Multiple Comparisons P values (2-tailed) test was used. For survival analysis, Peto & Peto modification of the Gehan-Wilcoxon test was used. P values less than 0.05 were considered significant.

## Results

### 3.1. Kinetics of the EPI encapsulation in the HSPC/Chol/DSPE-PEG 2000 (5.5:4:0.5) liposomes

**Figure 1:**
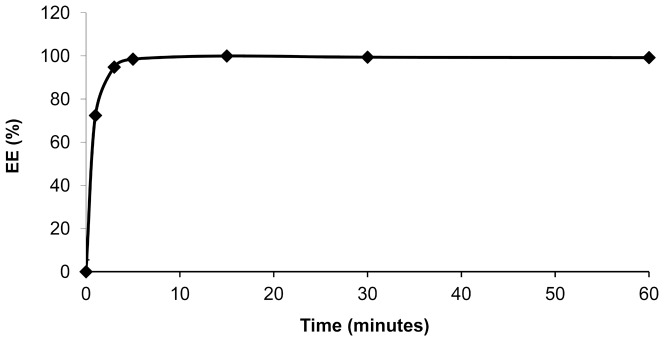
Kinetics of the EPI encapsulation in the HSPC/Chol/DSPE-PEG 2000 liposomes. The effect of incubation time of EPI on EE in 110/Chol/DSPE-PEG 2000 LUVs containing 300 mM EDTA diammonium solution, pH 4.3. The process was performed at 60°C and the drug-to-lipid ratio was 1:5 w/w. Errors bar are less than 5%,(n = 3).

To determine the time required for complete drug encapsulation within the liposomes, the EE of the EPI was measured at various time intervals. A drug-to-lipid ratio of 1:5 w/w and a temperature of 60°C were chosen for drug encapsulation as the high amount of the drug inside liposomes is correlated with better antitumor activity [25], [26]. Further increase in drug concentration could lead to liposome destabilization. As shown in Fig. 1, the kinetics of the process is very rapid. After just 2.5 min, the level of drug encapsulation was very high – about -95-97%. After 5–10 min the EE was almost 100% and no changes in the EE were observed during further liposome incubation at 60°C. Prolonged incubation of the obtained EPI-loaded liposomes for an additional two hours at 60°C did not lead to measurable back release of the drug from the liposomes (data not shown).

### 3.2. Cryo-TEM

**Figure 2:**
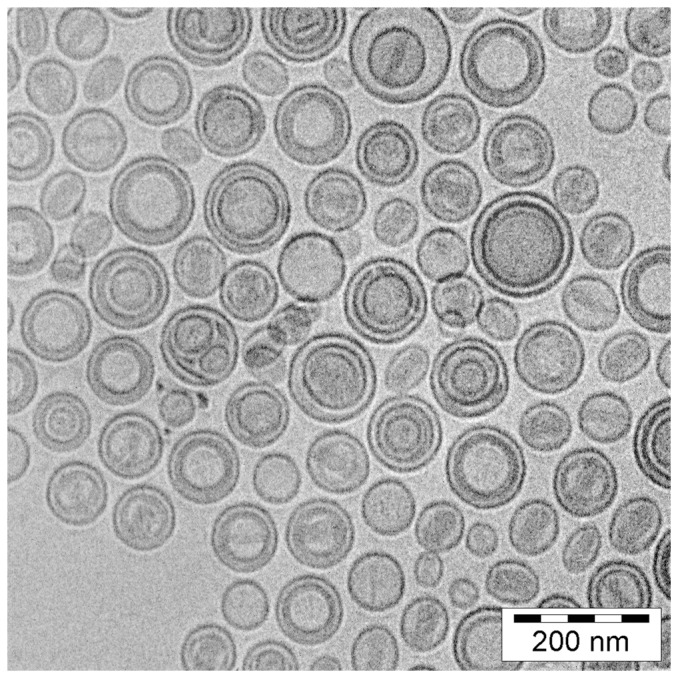
A Cryo-TEM study. A Cryo-TEM micrograph of HSPC/Chol/DSPE-PEG 2000 (5.5:4:0.5 mol/mol) liposomes loaded with EPI via an EDTA gradient at the 1:5 drug-to-lipid weight ratio. The internal buffer was 300 mM EDTA diammonium salt, pH 4.3. The external buffer was 150 mM PBS buffer, pH 7.4. Drug precipitates are seen as darker circular or button like structures inside the liposomes. The liposomes circular shape remains unchanged.

The Cryo-TEM technique was applied to visualize the physical state of the drug inside the liposomes. As shown in Fig. 2, drug precipitation was observed inside the HSPC/Chol/DSPE-PEG 2000 liposomes formed with EDTA gradient. Similarly to the case of drugs loaded via ammonium sulfate or citrate buffer gradients, EPI precipitates inside the liposomes, yet it forms a rather circular or button-shaped structures, not typical “coffee bean” structures as in case of DOX loaded liposomes by the ammonium sulfate method. The liposome shape remains unchanged.

### 3.3. The stability of the EPI-loaded liposomes in the presence of plasma

**Figure 3:**
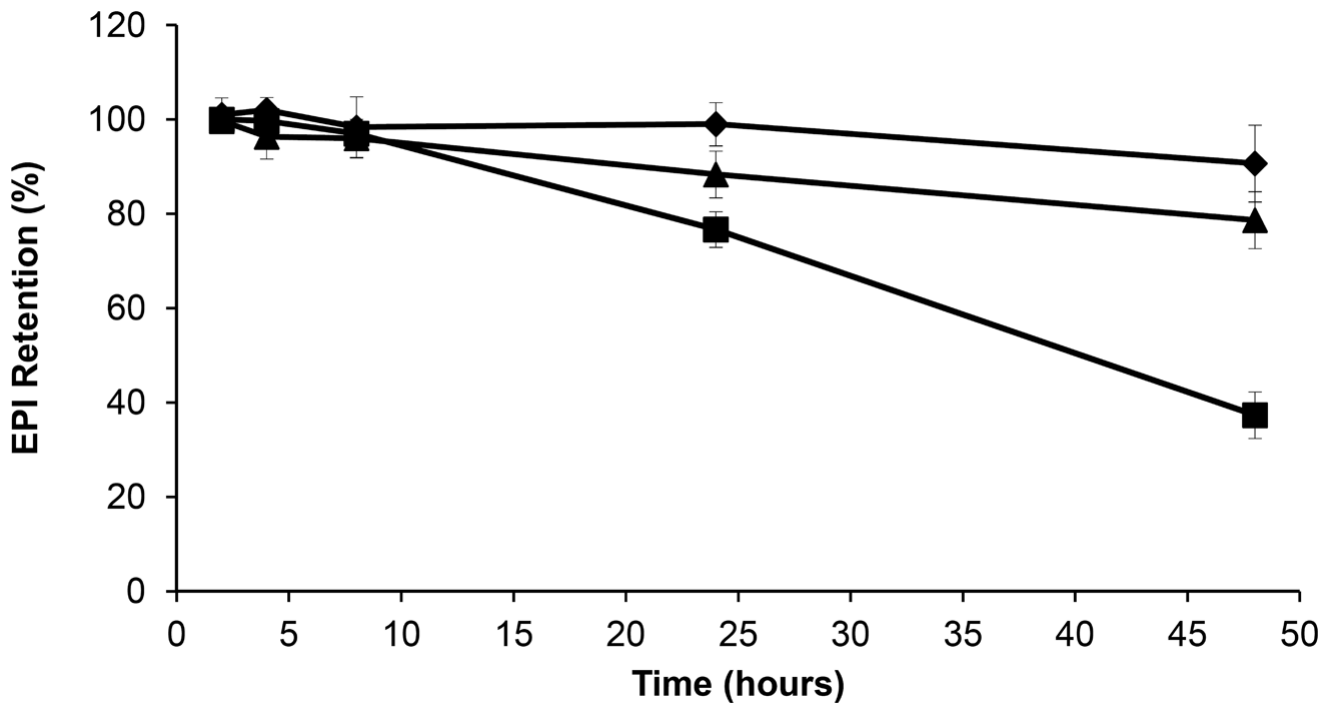
The in vitro stability of the EPI-loaded liposomes in the presence of plasma. Comparison of the plasma release of EPI encapsulated in HSPC/Chol/DSPE-PEG 2000 (5.5:4:0.5 mol/mol) liposomes via different ion gradients: (⧫) 300 mM EDTA diammonium salt solution, pH 4.3; (▴) 300 mM ammonium sulfate solution, pH 5.5; and (▪) 300 mM citrate buffer, pH 4.0. EPI loaded liposomes were mixed with fresh human plasma (1:1 v/v) and incubated for 48 h at 37°C. The bars represent the means +/− SD of three analyzes.

To assess and compare the stability of the EPI-bearing liposomes loaded via transmembrane gradients, three different liposomal preparations were studied. This comparison would also give information about the EPI retention properties under blood-like conditions of liposome formulations generated using the EDTA encapsulation method. HSPC/Chol/DSPE-PEG 2000 EPI-loaded liposomes were prepared as described in the Materials and Methods section. The drug release profile from the EPI-loaded liposomes in the presence of 50% human plasma is presented in Fig. 3. The Sepharose CL-4B columns were selected because it was not feasible to separate liposomes from plasma proteins and protein bond EPI which migrated as one fraction on Sephadex G-50 columns. Only free drug fraction could be separated that way. On Sepharose CL-4B columns the three different fractions could be collected – liposomal EPI, EPI bound to proteins and free drug.

For drug loading using the EDTA diammonium salt and ammonium sulfate gradient, only low drug leakage was observed over 48 h. More rapid drug leakage was observed in the case of liposomes where EPI was encapsulated via the citrate method, where about 60% leaked within 48 h. Although this experiment does not fulfill the requirements of sink conditions used to mimic in vivo liposomes leakage, it gives some insight on possible drug leakage differences in vivo.

### 3.4. Long-term retention experiments

**Figure 4:**
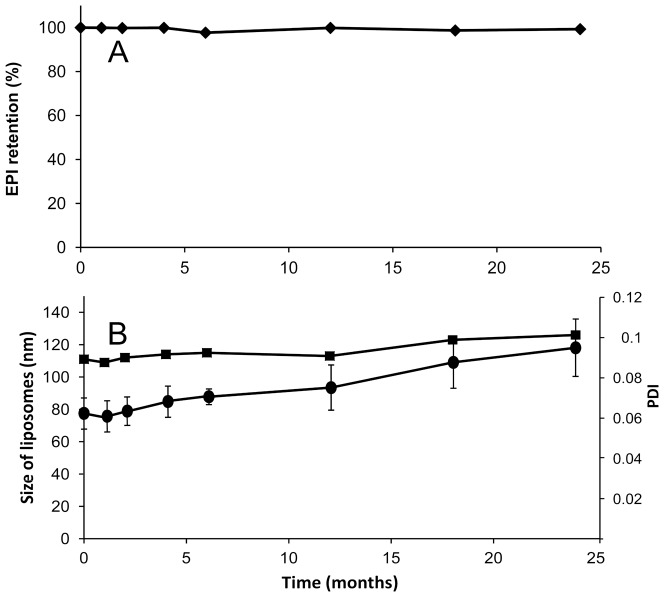
Long-term retention and stability experiments. The long-term stability of HSPC/Chol/DSPE-PEG 2000 (5.5:4:0.5 mol/mol) liposomes loaded with EPI via the EDTA ion gradient method and stored at 4°C for 24 months in PBS buffer at a drug concentration of 1.5 mg/ml illustrated in panel A as drug retention and in panel B as liposome size (▪, left axis) or polydispersity index changes (•, right axis). The bars represent the means +/− SD of three analyses. If not shown, then the errors bar were less than 5%.

Fig. 4A shows the long-term stability plot in terms of drug retention for liposomes loaded via the EDTA diammonium salt method at a drug-to-lipid ratio of 1:5 w/w. No significant drug leakage from the liposomal formulation stored in suspension was observed during two years of storage, indicating excellent EPI retention properties. Additionally, no essential changes in the mean size and polydispersity of the liposomes were noted in that time period as shown in Fig. 4B.

### 3.5. Plasma elimination of liposomal EPI

**Figure 5:**
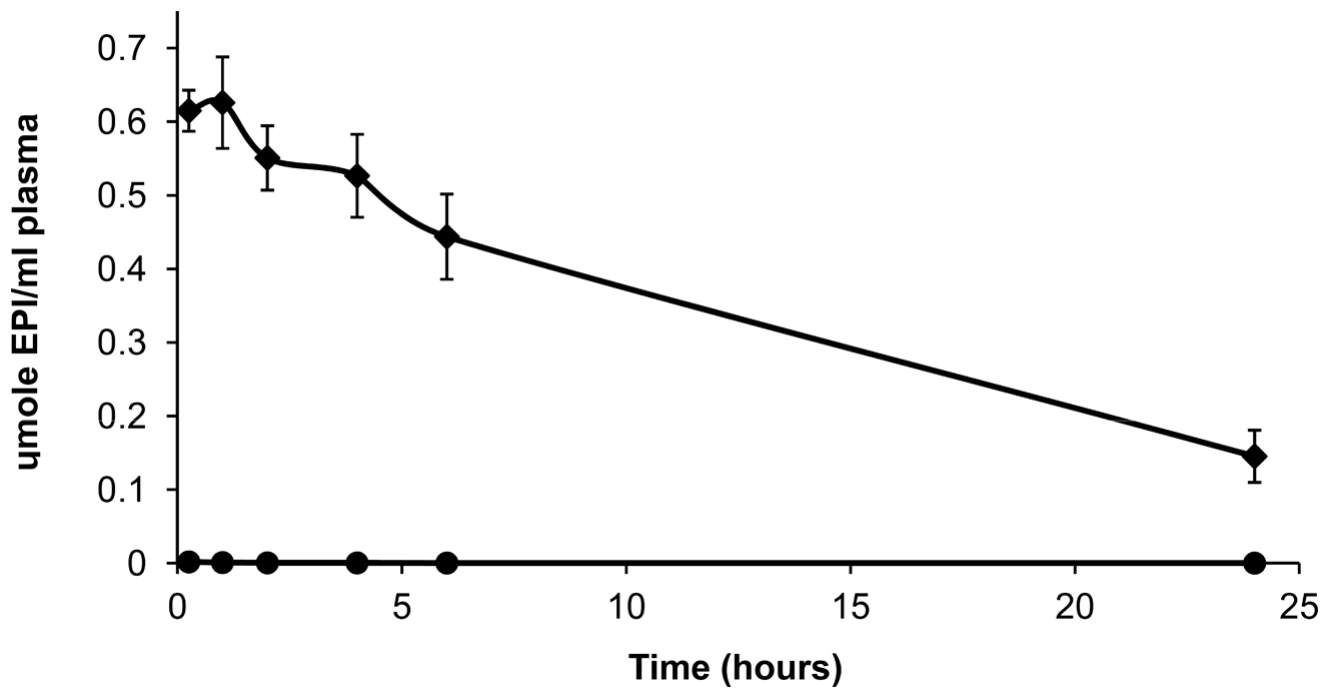
Plasma elimination of liposomal EPI. The plasma concentrations of EPI after the injection of the HSPC/Chol/DSPE-PEG 2000 (5.5:4:0.5 mol/mol) EDTA/EPI liposomes (⧫) and free EPI (•), into the mice at a dose of 34.5 μmole/kg body weight (20 mg/kg). The bars represent the means +/− SD of five analyses

Only less than one percent of the injected dose can be found in circulation 15 min after an intravenous injection of free EPI. This amount probably represents the protein-bound drug population, which is subsequently eliminated in a much slower manner (Fig. 5). The rest of the drug was already metabolized and its metabolites could be seen in the mouse urine, which showed red staining (not shown). In contrast to the free drug, as expected, the liposomal EPI is eliminated slowly with a calculated AUC of 7.648751 whereas the AUC for the free drug was only 0.009745 (Table 1).

**Table 1:** Summary of pharmacokinetic parameters of free and liposomal EPI.

Sample	AUC_0–24h_ (μmole h/ml)	AUMC (μmole h^2^/ml)	MRT (h)	Vd (ml/μmol)	T_1/2_ (h)	Clb (ml/kg/h)
Free EPI	0.0097	0.086	8.817	31214.815	6.111	3540.322
Liposomal EPI	7.648	64.490	8.431	38.030	5.844	4.510

Over 20% of the initial liposomal EPI dose is still present in the circulation 24 h after drug injection. The detailed data about both the free and liposome-encapsulated drug are given in Table 1. An open question at this point is whether this difference in the AUC of the free and liposome-encapsulated EPI also means a difference in the efficacy to inhibit tumor growth.

### 3.6. Human breast adenocarcinoma growth inhibition effected by the free and liposomal EPI

Long-circulating liposomes are known to accumulate inside tumors through the so-called EPR phenomenon, which is responsible for the increased leakage from the fenestrated blood vessels in the tumor tissues. Similarly, as already observed for other liposomal long-circulating drug formulations, the drug leakage and/or liposomal uptake by the RES system was slow enough to facilitate liposome accumulation inside the human breast tumor tissue in the mice. Depending on the liposome composition, drug bilayer interactions and encapsulation method, faster or slower drug release can be observed after liposomal drug accumulation inside the cancer tissue, resulting in a different antitumor activity for the drug. Therefore, we estimated the anticancer potential of the newly elaborated long-circulating liposomal formulation of EPI on the human MDA-MB-231 breast adenocarcinoma line, which was inoculated in NOD/SCID mice. On day six, the free and liposome-encapsulated EPI was injected into the mice at doses of 6 or 9 mg/kg body weight (3.47 and 5.12 μmole, respectively).

**Figure 6:**
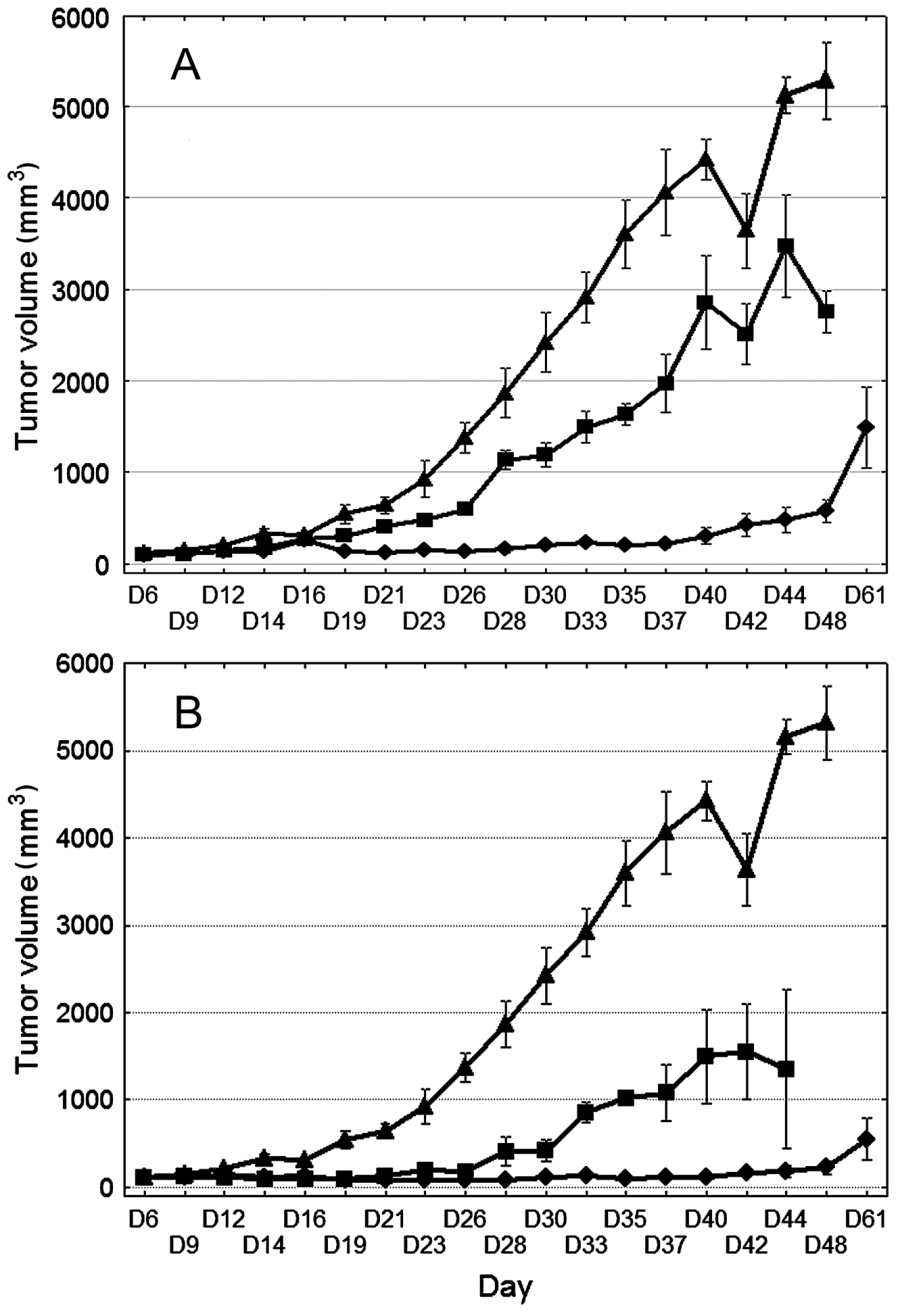
Human breast adenocarcinoma growth inhibition effected by the free and liposomal EPI. Effects of EPI (free or liposomal) on the MDA-MB-231 breast adenocarcinoma model given at a dose of 6 (panel A) or 9 (panel B) mg/kg. (▴) Control, saline injected mice, (▪) mice given free EPI, and (⧫) mice given liposome-encapsulated EPI. The bars represent the means +/− SD (n = 9).

**Figure 7:**
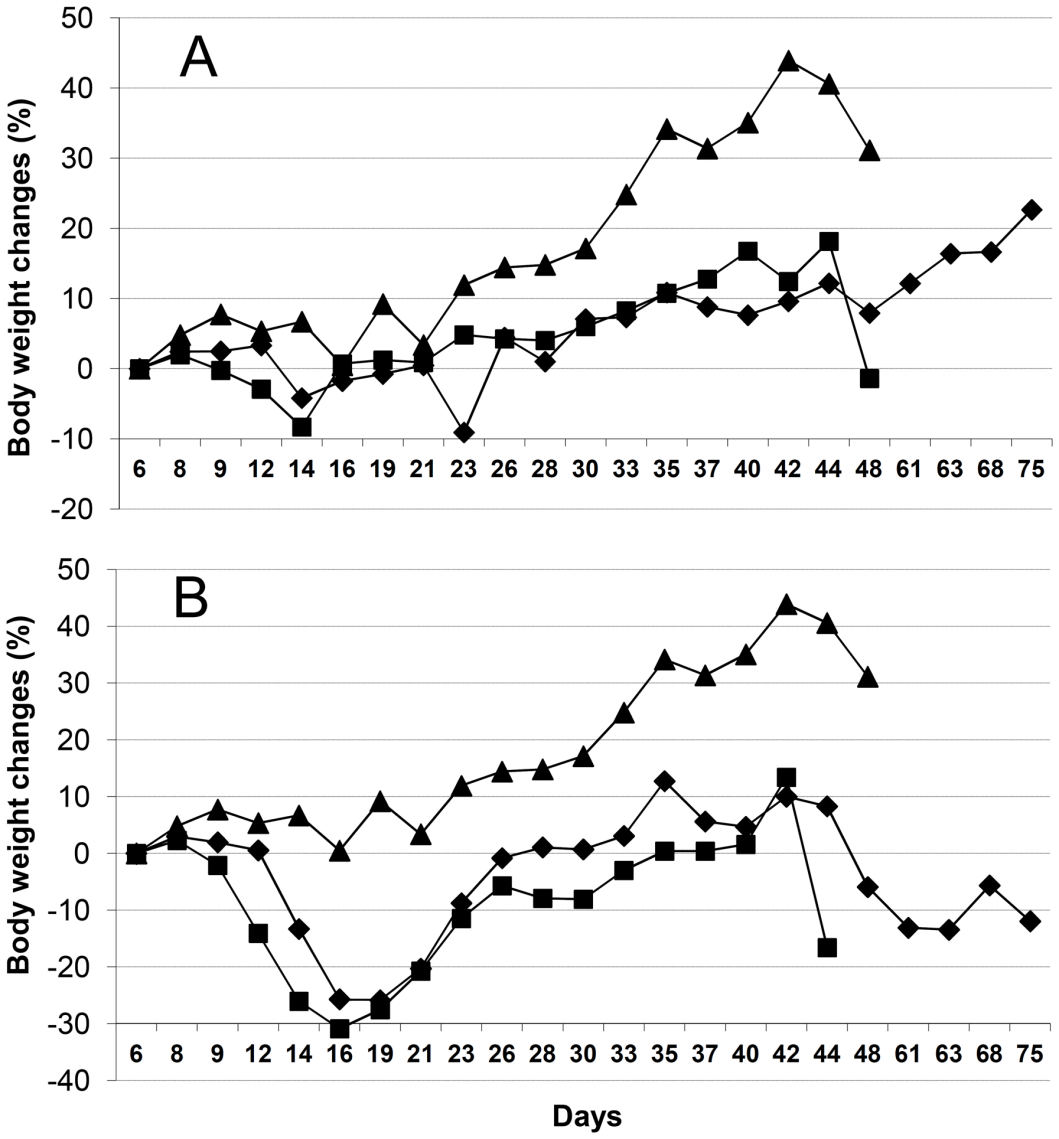
Body weight changes. Effect of the free and liposome-encapsulated EPI given at a dose of 6 (panel A) or 9 (panel B) mg/kg on the body weight of (▴) control, saline-injected mice; (▪) mice given free EPI; and (⧫) mice given liposome-encapsulated EPI.

The liposomal formulation of EPI administered at 9 mg/kg significantly inhibited tumor growth as compared to the results for the control group from day 14 to 48 of observation. Free EPI used at the same dose in a parallel experiment significantly retarded tumor growth only from day 14 to 23 (p<0.05, non-parametric Kruskal-Wallis ANOVA followed by Multiple Comparisons p values, Fig. 6b) and the toxicity of such treatment was high (body weight loss of 30%, Fig. 7b). Moreover, 5 of the 8 mice treated with free EPI died by day 16 of the experiment.

The most interesting results were those obtained with the use of the lower dose of EPI (6 mg/kg). At this dose, free EPI revealed only low toxicity (body weight lost did not exceed 10%, Fig. 7a), but its activity was only modest (Fig. 6a).The liposomal formulation of EPI used at a dose of 6 mg/kg showed significant and prolonged antitumor activity (Fig. 6a). A statistically significant retardation of tumor growth was observed in this group of mice from day 19 to 37 as compared to the control, whereas the antitumor activity of the free EPI was not statistically significant. The median survival time of the control mice was 47 days, and that of the mice treated with 9 or 6 mg/kg of the liposomal formulation of EPI was 68 and 56 days, respectively; in these two groups, one mouse was still alive at the end of the experiment, but it was excluded from the calculation (Table 2). On day 42, the animals of the control group with higher tumor volumes died. This meant the mean tumor volume decreased. This had a visible influence on the tumor volume plot in the control group (Fig. 6A and 6B).

**Table 2:** Survival time of mice treated with free or liposomal EPI at a dose of 6 or 9/kg.

*Group*	*Median survival time SD*	*ILS%*	*N*
Control	47	-	8
Free EPI 6 mg/kg	49	3	8
Free EPI 9 mg/kg	16	−66	8
Liposomal EPI 6 mg/kg	56	19	7
Liposomal EPI 9 mg/kg	68*	45	7

N – number of mice per group, *p<0.05 as compared to the results for mice treated with 9 mg/kg free EPI (Kruskal-Wallis multiple comparison test). ILS – Increased Life Span.

**Figure 8:**
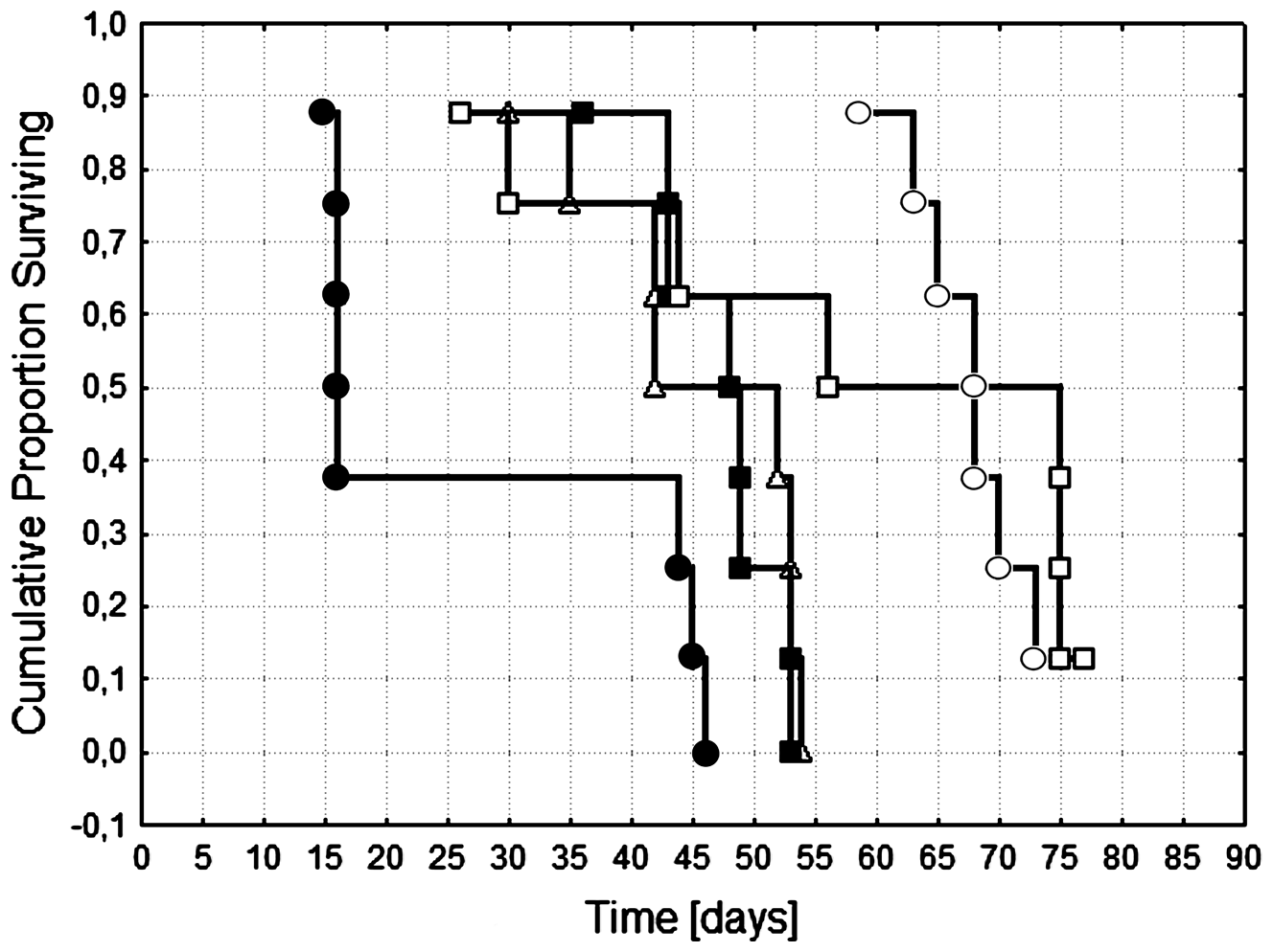
Survival analysis of mice bearing MDA-MB-231 tumors. (▵) control, saline-injected mice; (▪) mice given 6 mg/kg free EPI or (•) 9 mg/kg free EPI; and (□) mice given 6 mg/kg liposome-encapsulated EPI or (○) 9 mg/kg liposome-encapsulated EPI.

Analyzing the increase in life span (ILS), we can conclude that the liposomal formulation of EPI can prolong the survival of mice not only due to the prolonged effect of tumor growth retardation, but also by decreasing the toxicity of EPI. This is especially clear in the case of the mice that received a dose of 9 mg/kg, where high toxicity of free EPI was observed (mice died before those from the control group; Table 2). The survival analysis using the Peto & Peto Wilcoxon test shows that a statistically significant prolongation of survival was observed for the mice given 9 mg/kg of the liposomal formulation of EPI compared to the control group (Fig. 8).

## Discussion

In this study, the efficacy of a new liposomal formulation of EPI was compared to that of the free form of EPI on a human breast MDA-MB-231 cancer xenograft model. Liposomes, as one of the best available drug delivery systems, were selected because of their rare characteristics, including their long half-life in the blood, their high drug payload, and their ability to extravasate from the circulation into the tumor site. EPI was selected since it has better properties than DOX, which is also used in breast cancer treatment. EPI is less toxic at the same activity level, so about 30% higher doses can be administered offering a higher therapeutic index than with DOX.

As mentioned, besides these very interesting drug properties, only a limited number of studies have been conducted on liposomal formulations of EPI and on their properties compared to those of liposomal formulations of DOX. To the best of our knowledge, there have been no tests on a breast cancer model, which seems to be the primary target for liposome-encapsulated EPI [27]. Therefore, our goal was the development of a stable and efficient liposomal formulation of EPI.

A new anthracycline encapsulation method was selected for this purpose. It was recently used for idarubicin encapsulation [19]. The method uses the EDTA ion gradient as a driving force, enabling efficient drug accumulation inside the lipid vesicles, and it yields high encapsulation efficiencies and remarkably increased *in vivo* stability for fast-leaking hydrophobic anthracyclines, such as IDA. This is thanks to a very low solubility of the IDA-EDTA salt. In the case of EPI or DOX, the drug EDTA salts have better solubility at pH 4–5 than EPI-sulfate salts (0,145 vs 0,34 mg/ml for EPI sulfate and EDTA salts, respectively, data not shown) so according to this solubility test, an increased part of drug population should be in a soluble, not precipitated state inside drug-loaded liposomes when EDTA method is applied, compared to ammonium sulfate method. This drug population leaks first when the liposomes are destabilized by plasma proteins, lipid degrading enzymes involved in pH gradient collapse and pore formation in the bilayer. Additionally, as seen on the Cryo-TEM images, EPI-EDTA precipitates formed inside the liposomal vesicles have a circular- and button-like rather than elongated bundle structure which is observed in the DOX formulation loaded by ammonium sulfate method. This can contributes to faster drug leakage from the liposomes after liposome destabilization by mentioned factors by increasing the effective drug precipitate surface. The difference between EPI-EDTA and EPI-sulfate precipitate structures was already observable during the solubility test. The EPI-sulfate precipitate was dense and sedimented easily while EPI-EDTA salt had a rather viscous, syrup-like appearance and was difficult to sediment (data not shown) and also for above mentioned reasons drug dissolution rate should be faster for EDTA formulation. Therefore the EDTA loading method seems to have an advantage in this aspect. In the case of EPI, EDTA ion gradient method ensures practically 100% drug encapsulation efficiency (EE) at a drug-to-lipid ratio of 1:5 (Fig. 1), and similarly high EE were measured for drug-to-lipid ratios of 1:4 and 1:3. At a drug-to-lipid ratio of 1:2, the EE decreases to about 65% and the liposomes became unstable(data not shown). This formulation is characterized by very good long-term stability in suspension. During two years of storage at 4°C, very low drug leakage was observed. To explore the formulation potential in terms of drug release in harsher, blood-like conditions, three liposomal EPI formulations differing only in terms of their encapsulation method (EDTA, ammonium sulfate and citrate) were compared. The EDTA and ammonium sulfate methods gave similarly stable liposomes with low EPI leakage, whereas citrate-derived liposomes displayed 60% leaking during the 48 h of experimental observation. The ammonium salt used for the sulfate and EDTA liposomes ensures much better pH gradient stability derived by the internal free ammonia-generating mechanism, which lowers internal pH after the pH gradient destabilization. That can explain the reported differences. Additionally, in the case of the citrate buffer, the drug precipitate is not as stable as in the other applied gradients, so the drug is released faster after liposome bilayer destabilization and pH gradient collapse that result from interactions with the plasma components. A proper leakage rate (albeit not too rapid) is essential for maintaining the formulation activity since drug-loaded liposomes have to accumulate in the tumor and induce anticancer activity.

The most interesting aspect was related with the antitumor activity of the formulations. 24 h after the injection of the liposomal formulation of EPI, almost 25% of the injected dose was still present in circulation. This gave the possibility that liposomes could accumulate passively inside the tumor tissue through the EPR effect. We suppose, as is observed in the case of long-circulating DOX-encapsulating liposomes, that the measured drug concentration represents the mostly liposome-encapsulated EPI population, which is not available for normal, healthy tissues like those of the heart or kidney, meaning the liposome-encapsulated EPI was expected to have less severe side effects. Indeed, the antitumor activity of the liposomal formulation is superior to that of the free drug. For the higher tested dose of 9 mg/kg body weight, the liposomal EPI showed much better anticancer activity with significant inhibition of tumor growth compared to the control group from day 14 to 48 of observation and the free EPI-treated group from day 14 to 23. Simultaneously, it displayed decreased overall toxicity. As mentioned in the Results section, 5 out of 8 mice treated with free EPI at a dose of 9 mg/kg died by day 16 of the experiment. In the case of the same dose of liposomal EPI, the mice started to die from day 60 on, from late drug toxicity and tumor regrowth. At the lower tested dose of 6 mg/kg, the performance of the liposomal formulation of EPI was also superior. Free EPI activity was in this case statistically not significant, whereas the liposomal formulation of EPI exhibited significant tumor grow retardation from day 19 to 37 as compared to the control group. The toxicity of both forms was low and the mice in the free drug group started to die from day 37 on because of the huge tumor mass. This did not correlate with the drug toxicity, as seen in Fig. 7A – there was no essential decrease in body mass observed with the free or liposome-encapsulated drug at the 6 mg/kg dose. The most important observation is that while the free drug does not kill the animals at a concentration of 6 mg/kg, the liposomal formulation is still much more active and in fact the free form is not statistically better than saline alone.

The advantages of this liposomal formulation arise from the potential to administer it at much higher doses with moderate toxicity and much higher activity. The liposomal formulation of EPI given at the higher tested dose of 9 mg/kg body weight increased median survival time from 47 to 68 days (Table 2). At the same time, the median survival time for the free drug group at the same dose was only 16 days. That indicates some decrease in drug toxicity upon liposomal encapsulation.

Similar observations were made for the liposomal formulation of EPI administered to mice bearing the mouse colon 26 tumor, indicating that the liposomal formulation of EPI was at least as toxic or less toxic than the free drug, and at milligram-equivalent doses, PEG-liposome-encapsulated EPI was more active than the free drug [17].

Liposomes are regarded as one of the best available drug delivery systems. Because a high drug-to-lipid ratio is possible when active drug-loading methods are applied, an essential drug amount can be deposited near tumor cells when the liposomes reach the direct environment of the tumor thanks to the so-called EPR effect. To achieve this, the liposome must circulate for enough time to reach the tumor and it must retain its payload until needed. The size of liposomes must be roughly 100 nm or less, but some researchers claim that the smaller the drug vehicle, the better the tumor penetration efficiency [28]. Therefore, the size of liposomes should possibly be decreased to about 40–60 nm to achieve better anticancer activity. The next step in studies of liposome-encapsulated EPI should be to compare those larger and smaller liposome versions to see if the potential of this formulation can be increased further.

Usually, when liposomes are used as anticancer drug carriers, decreased drug side effects are noted. This correlates with the slow drug release from the liposomes in circulation. No free drug high peak is observed, but liver-accumulated liposomes are slowly degraded until some amounts of the drug are liberated into the blood stream. This is responsible for the late EPI toxicity observed on the group receiving 9 mg/kg liposome-encapsulated EPI (Fig. 7B). This is difficult to avoid, especially in an animal model, but in the case of humans, this effect should be less pronounced than that observed in mice.

## Conclusions

The reported liposomal EPI formulation exhibits very good long-term stability behaviour and antitumor activity against MDA-MB-231 human breast adenocarcinoma. These results support the rationale behind the preparation of a liposomal form of EPI and indicate its possible practical application.
